# Development and validation of an artificial intelligence-assisted system for automatic Boston scoring of bowel cleanliness in colonoscopy (with video)

**DOI:** 10.3389/fpubh.2025.1708325

**Published:** 2026-01-02

**Authors:** Jian Chen, Jingzhi Xu, Kaijian Xia, Qiuwen Hua, Xiaodan Xu, Ganhong Wang

**Affiliations:** 1 Department of Gastroenterology, Changshu Hospital Affiliated to Soochow University, Suzhou, Jiangsu, China; 2 Changshu Key Laboratory of Medical Artificial Intelligence and Big Data, Suzhou, China; 3 Department of Gastroenterology, Changshu Hospital Affiliated to Nanjing University of Chinese Medicine, Suzhou, Jiangsu, China; 4 Center of Intelligent Medical Technology Research, Changshu Hospital Affiliated to Soochow University, Suzhou, China

**Keywords:** artificial intelligence, YOLO, colonoscopy, Boston bowel preparation scale, bowel cleanliness

## Abstract

**Background:**

Bowel cleanliness is a critical factor affecting the detection of adenomatous polyps and early tumors. The Boston Bowel Preparation Scale (BBPS), a widely used evaluation tool, has limitations, including interobserver variability and insufficient standardized training. This study aims to develop an artificial intelligence-driven automatic BBPS scoring and teaching system.

**Methods:**

Colonoscopy image and video data were collected from three centers between June 2019 and August 2024, categorized into different BBPS scores (0, 1, 2, 3), ileocecal part, and instrument operation frames. Transfer learning and fine-tuning were performed on four pre-trained YOLOv11 models. Performance metrics included accuracy, precision, sensitivity, and AUC. Grad-CAM was used to provide visual explanations of the best-performing model, which was further developed into a system capable of real-time and cumulative BBPS assessment for every video frame.

**Results:**

Among the four models, YOLOv11m performed the best, achieving an accuracy of 99.86%, precision of 99.74%, sensitivity of 99.74%, and an F1 score of 99.75% on the validation set. On the test set, the model attained a weighted average precision of 95.37%, specificity of 98.25%, and an AUC of 0.996. Based on this model, the AutoBBPS system was developed, which automatically initiates real-time cumulative BBPS scoring once the cecum is reached. In image-level human-machine comparison experiments, the system outperformed junior endoscopists in recognition accuracy and was comparable to senior endoscopists. Video-level human-machine comparison experiments further evaluated the accuracy of the AutoBBPS system against endoscopists under varying confidence thresholds.

**Conclusion:**

The AutoBBPS system, developed using YOLOv11, provides real-time and cumulative BBPS scoring for every video frame, effectively assisting endoscopists in improving scoring efficiency and accuracy. Additionally, the intelligent BBPS teaching assistant is particularly beneficial for junior endoscopists, promoting standardized training and enhancing overall scoring quality.

## Introduction

The large intestine, also known as the colon, constitutes the lower segment of the human gastrointestinal tract and is highly susceptible to severe conditions such as cancer and chronic inflammation. Colorectal cancer ranks as the third most common cancer globally, with approximately 1.93 million new cases and 930,000 deaths reported in 2020, a number projected to rise to 3.2 million cases and 1.6 million deaths by 2040 (1). Currently, colonoscopy is the gold-standard recommendation for the diagnosis and screening of colorectal diseases (2). The effectiveness of colonoscopy largely hinges on adequate bowel cleanliness, which ensures a clear view of the intestinal mucosa. The adenoma detection rate is inversely correlated with the risk of colorectal cancer and has been demonstrated to be closely linked to bowel cleanliness (3, 4). As such, bowel cleanliness is widely regarded as a reliable quality indicator for colonoscopy.

The Boston Bowel Preparation Scale (BBPS) is currently the most validated and widely used scoring system for assessing bowel preparation in colonoscopy procedures both domestically and internationally. However, BBPS relies on the subjective memory of endoscopists post-procedure, making it susceptible to evaluator bias and recall errors. This limitation is particularly pronounced during challenging colonoscopies or when performing intricate instrument operations, which can significantly prolong the duration of the procedure. Consequently, the development of an objective and automated AI-assisted bowel cleanliness assessment system is of critical importance. Such a system would alleviate the operational burden on endoscopists, enhance scoring consistency, and optimize the utilization of medical resources.

In recent years, the rapid advancement of artificial intelligence (AI) has led to its extensive application in the medical field, including aiding in diagnostic tasks such as CT imaging, gastrointestinal endoscopy, and the identification of traditional Chinese medicinal herbs (5–7). AI has demonstrated advantages such as high detection speed, superior accuracy, enhanced objectivity, and cost-effectiveness. If AI can achieve automated and accurate BBPS scoring, it would alleviate the burden on physicians during procedures, allowing them to focus more on detecting polyps and early-stage cancers. Moreover, it would reduce inconsistencies in scoring results caused by subjective evaluator judgment. In this study, we developed a deep learning-based automated BBPS scoring system along with an intelligent teaching assistant, providing an objective and standardized AI-driven method for assessing bowel cleanliness during colonoscopy.

## Methods

### Datasets

**Figure 1:**
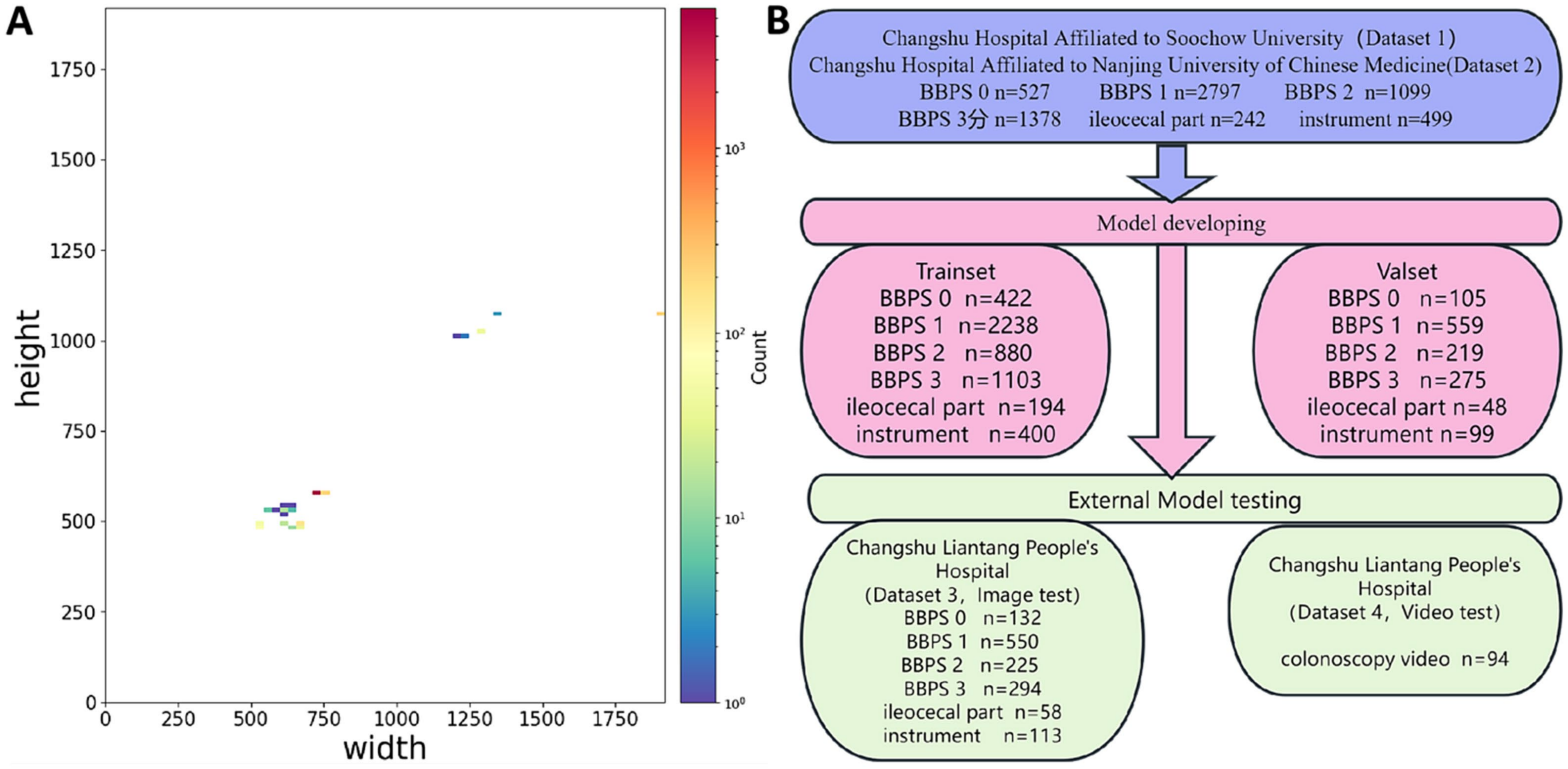
Analysis of image characteristics in the dataset. **(A)** Distribution of image dimensions. Red indicates a higher number of images for a given dimension, while blue represents fewer images. The dataset includes various image dimensions, with the two most common being 720 × 576 pixels and 660 × 497 pixels. **(B)** Distribution of images across categories.

**Figure 2:**
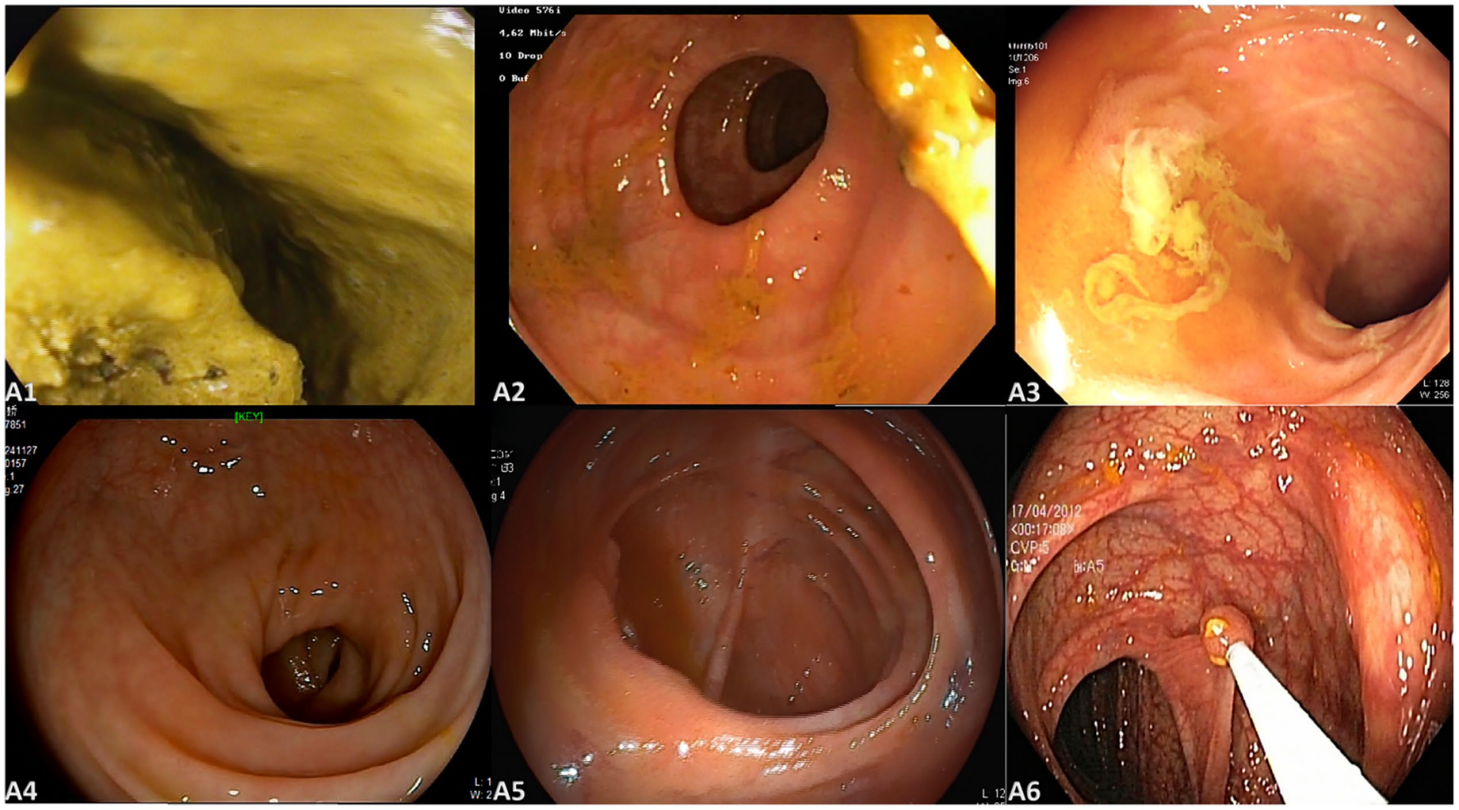
Representative images from the dataset; **(A1–A4)** represent BBPS scores of 0, 1, 2, and 3, respectively; **(A5)** is an image of the ileocecal part; **(A6)** is an image of instrument operation.

This study utilized four datasets spanning from June 2019 to August 2024, comprising a total of 7,914 images and 94 videos. Dataset 1 and Dataset 2, sourced from Changshu Hospital Affiliated to Soochow University and Changshu Hospital Affiliated to Nanjing University of Chinese Medicine, respectively, included 6,542 colonoscopy images used for model training and validation. Dataset 3, provided by Changshu Liantang People’s Hospital, contained 1,372 images and served as the image test set. Dataset 4, also from Changshu Liantang People’s Hospital, included 94 colonoscopy videos designated for the video test set. The test sets were used solely to evaluate model performance without participating in training or parameter tuning, ensuring their independence. A feature analysis of the dataset images is presented in Figure 1. The image categories included varying bowel cleanliness scores (BBPS scores of 0, 1, 2, and 3), images of the ileocecal region, and those depicting instrument operations. Representative images are shown in Figure 2. The three medical centers employed colonoscopy equipment from three different manufacturers, including five SonoScape HD-550 systems (China), seven Olympus CV-V1 systems (Japan), and two Pentax EPK-i7000 systems (Japan).

### Image annotation

This study employed the Boston Bowel Preparation Scale (BBPS) to assess bowel cleanliness, as it is currently the most validated and widely utilized scoring system in large comprehensive hospitals (8). BBPS divides the colon into three parts: the right colon, transverse colon, and left colon, and evaluates the cleanliness of each part separately. The scoring criteria are as follows: when severe fecal residue completely obstructs the view, the score is 0; when the mucosa is mostly covered by feces, making observation difficult, the score is 1; when part of the mucosa is visible, the score is 2; when the cleanliness is excellent and the mucosa is clearly visible, the score is 3. The total score ranges from 0 to 9, with 9 indicating excellent bowel cleanliness. Higher scores represent better cleanliness.

**Figure 3:**
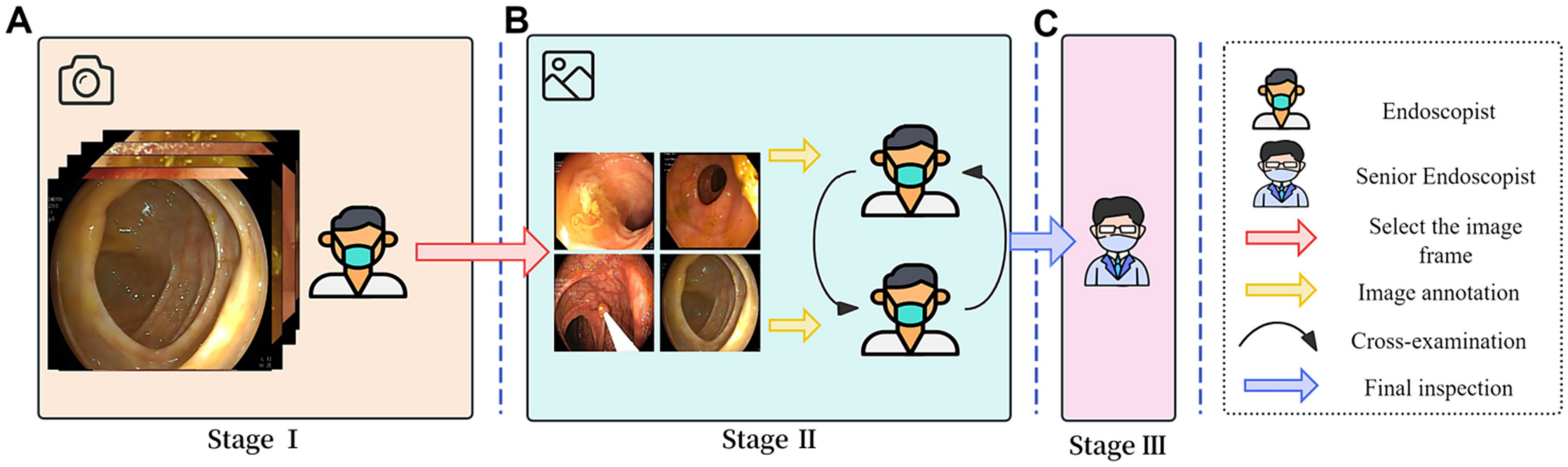
Image annotation process.

The image annotation process in this study was divided into three stages, with each stage managed by a different team of endoscopists. Before commencing the annotation work, the teams underwent multiple training sessions on BBPS-related theory and practice. In the first stage, endoscopists selected video segments of interest and converted them into single-frame images. In the second stage, two groups of endoscopists screened the image frames, retaining clear images and those containing various categories of lesions, while performing cross-checks. In the third stage, senior endoscopists reviewed the annotation results and made the final decisions. Figure 3 provides a detailed depiction of the image annotation process.

### Image preprocessing

To enhance the generalization ability of the training model, we implemented a standardized, online image preprocessing and data augmentation pipeline. During preprocessing, all images were letterboxed to 640 × 640 while preserving the original aspect ratio (padding applied as needed). For augmentation, we applied: (1) random horizontal flip with probability 0.50; (2) RandomResize with a scale range of 0.90–1.10 relative to the shorter side and aspect-ratio jitter of ±10%; (3) RandomCrop with a crop ratio of 0.80–1.00 of the resized image (crops < 0.80 were rejected to avoid excessive content loss); and (4) HSVRandomAug (YOLO built-in) with hue ±0.015 (9), saturation ±0.70, and value ±0.40. All operations were performed online per image and per iteration within the training loop (10), eliminating the need to pre-generate or store augmented images while ensuring the model encountered slightly varied samples in each epoch.

### Model training configuration

**Figure 4:**
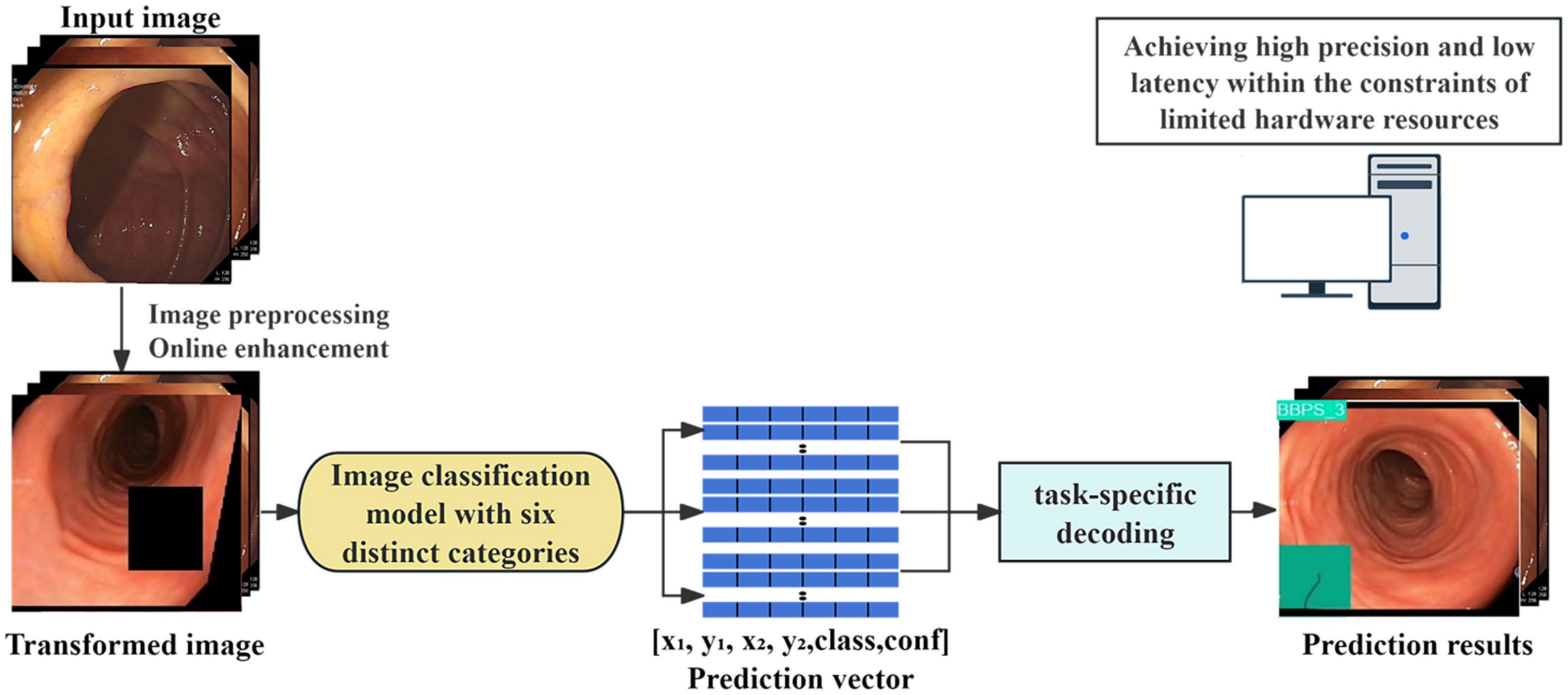
Development of an artificial intelligence model for automatic recognition of colonoscopy images.

This study employed a transfer learning strategy (11), selecting four YOLOv11 models of different scales pre-trained on the ImageNet dataset (12): nano (n), small (s), medium (m), and large (l). These versions represent varying model sizes and complexities, and the training workflow is illustrated in Figure 4. During training, pre-trained weights were loaded, and all layers were fine-tuned on the dedicated dataset constructed for this study. The optimizer was automatically selected, and the learning rate was adjusted based on the configuration file to optimize training performance. The study set a maximum of 100 epochs for training, with a batch size of 32.

Performance evaluation metrics included accuracy, precision, recall, and F1 score, calculated and recorded based on the predictions of the validation set. To improve training efficiency, automatic mixed precision training was enabled on the graphics processing unit (GPU). An early stopping mechanism was introduced, with a patience value of 5, meaning training was terminated early if validation performance showed no improvement for 5 consecutive epochs to prevent overfitting. All operations were conducted within the PyTorch framework.

### Development of a fully automated BBPS system

**Figure 5:**
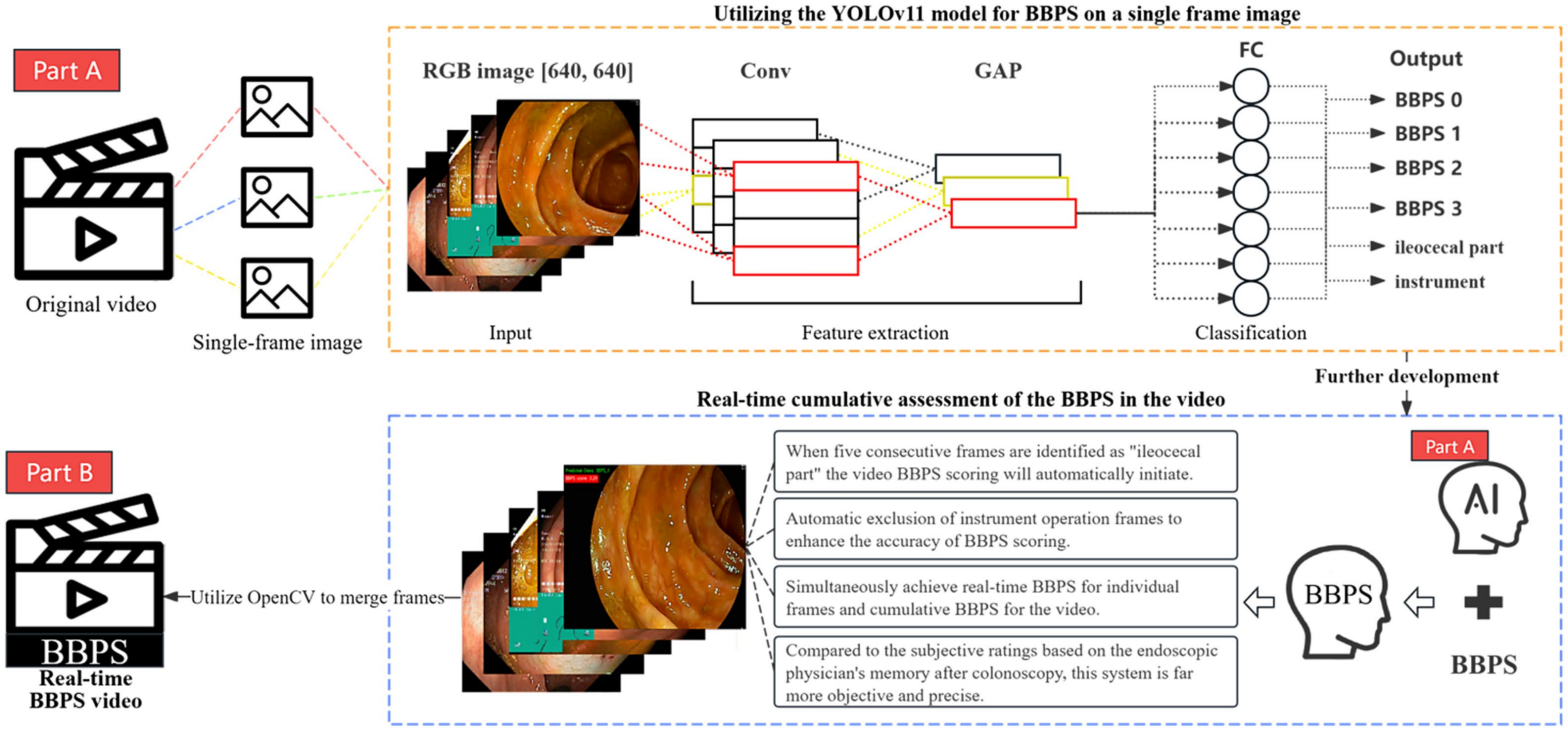
Schematic diagram of the automatic Boston scoring system architecture.

To ensure the robustness and practical applicability of the model, the performance of four YOLOv11 variants was compared, and the best-performing model was selected. This model was then utilized for real-time inference and prediction on dynamic colonoscopy videos. During the prediction process, OpenCV technology was integrated to analyze colonoscopy videos frame by frame, enabling real-time BBPS scoring for individual frames as well as cumulative BBPS scoring for entire videos. The system leveraged OpenCV for video input and output, real-time annotations, and score display. Based on the model’s predicted categories (e.g., “BBPS 0,” “BBPS 1,” “BBPS 2,” “BBPS 3,” “ileocecal part,” and “Instrument”), it detected whether the ileocecal region had been reached and counted the frame numbers for each scoring category. Additionally, the system excluded frames involving instrument operation to further enhance scoring accuracy. The architecture of the developed system is illustrated in Figure 5.

In the specific implementation, once the system detects the arrival of the ileocecal region, it begins to count the predicted categories for each frame. Using the “defaultdict” class from Python’s collections module, it records the number of frames in which each category appears, along with the total frame count. Subsequently, the scores are weighted according to the weights of each category, dynamically generating a cumulative BBPS score, which is then displayed in real-time on the video frames. Finally, the system exports the processed results as a newly annotated video file for verification and clinical application. We have named this system AutoBBPS. The scoring formula is as follows:


$$\begin{array}{l} \text{BBPS score}=9\times (\frac{\text{Frames with BBPS score}\;3}{\text{Total frame count}-\text{instrument frame count}})+\\ 6\times (\frac{\text{Frames with BBPS score}\;2}{\text{Total frame count}-\text{instrument frame count}})+\\ 3\times (\frac{\text{Frames with BBPS score}\;1}{\text{Total frame count}-\text{instrument frame count}}) \end{array}$$

### Model interpretability analysis

The high computational cost, difficulties in data acquisition, and the “black-box” nature of deep learning methods have limited the widespread application of computer vision in the medical field. To address these challenges, Explainable AI (XAI) technologies have emerged, aiming to enhance the transparency of models. This study employs Grad-CAM to reveal critical image regions in the model’s decision-making process by generating heatmaps (13). In the specific implementation, a model wrapper, MyModelWrapper, is first defined to adapt the output of the YOLOv11 model. The model is then loaded and wrapped, with the penultimate layer selected as the target layer. By checking the device and moving the model to the GPU, Grad-CAM is initialized, and a list of images for inference is obtained. For each image, it is read, converted to RGB, resized to 224 × 224 pixels, normalized, and tensorized, with gradient computation enabled. After the model predicts the category, Grad-CAM is used to generate a grayscale heatmap, which is then overlaid onto the original image to produce a visual result. Finally, the original image, heatmap, and overlaid image are saved, and the processing status is output. Through this workflow, the heatmaps generated by Grad-CAM help researchers and clinicians understand the basis of the model’s decisions, verify whether the model focuses on key features in medical images, and enhance the trustworthiness and application value of AI in the medical field.

### Terminal deployment

This study converted the PyTorch-based AutoBBPS system into the Open Neural Network Exchange (ONNX) format to enable efficient cross-platform (e.g., Linux, Windows, macOS) and multi-hardware (e.g., CPU, GPU) operation. To provide a standardized BBPS intelligent teaching tool and minimize scoring deviations among endoscopists caused by subjective judgments and experience differences, a web application (App) was developed using the Streamlit framework (v1.37.0) (13, 14). The application integrates the ONNX model and supports image, video, and real-time camera inputs.

The application features a modular sidebar layout, allowing users to upload images or videos, select sample images, or perform real-time BBPS scoring using a camera. By integrating PIL and OpenCV, the system ensures consistent model inputs and accurate predictions through automatic orientation adjustments and size standardization of input images. The ONNX model is dynamically loaded, and upon clicking the “Predict” button, the app quickly outputs the top five predicted categories along with their confidence scores. The category with the highest confidence is overlaid on the image, providing intuitive and accurate classification results. This design significantly enhances usability and user experience.

### Human-machine comparison

This study conducted evaluations at both the image and video levels through human-machine comparative experiments. At the image level, two senior endoscopists (with over 5 years of colonoscopy experience) and two junior endoscopists (with less than 3 years of experience) were invited to independently assess a test set of images (*n* = 1,372). The results of their evaluations were compared with predictions made by the AI system using four different YOLO models, focusing on differences in diagnostic accuracy and evaluation speed. At the video level, the AutoBBPS system, developed based on the best-performing model, was used to independently score BBPS on 94 videos (Dataset 4). The system’s performance was compared with that of the four endoscopists, analyzing prediction accuracy at different confidence levels. On the validation set, we swept decision thresholds from 0.50 to 0.95 (step 0.05) and selected 0.80 based on the highest agreement with senior endoscopists (Cohen’s *κ*) and a stable precision-recall trade-off.

### Experimental platform and evaluation metrics

The experimental environment for this study was built on a high-performance computing platform with the following hardware configuration: an NVIDIA GeForce RTX 4080 SUPER GPU (16GB VRAM), an Intel (R) Core (TM) i7-14700K processor (3.4 GHz base frequency), 32GB of RAM, and 1.9 TB of SSD storage. In terms of software, the PyTorch framework (version 2.5.1) was used for AI model development and training, while the OpenCV library (version 4.10.0.84) was employed for image data processing. To enhance data processing efficiency and visualization quality, the team integrated data analysis tools such as Pandas (version 2.2.3), NumPy (version 2.0.2), Matplotlib (version 3.9.2), and Plotly (version 5.16.1). Additionally, the Weights & Biases (wandb, version 0.18.7) platform was utilized for real-time monitoring and visualization of the experimental process, ensuring data traceability and providing clear comparability of experimental results.

The performance of the AI system is comprehensively evaluated using multiple metrics, including Sensitivity, Specificity, Precision, Accuracy, F1 Score, Average Precision (AP), Area Under the Curve (AUC), and Weighted Average. The calculation formulas are as shown in Equations 1–8.


(1) $$\text{Sensitivity}=\frac{\mathrm{TP}}{\mathrm{TP}+\mathrm{FN}}$$

(2) $$\text{Specificity}=\frac{\mathrm{TN}}{\mathrm{TN}+\mathrm{FP}}$$

(3) $$\text{Precision}=\frac{\mathrm{TP}}{\mathrm{TP}+\mathrm{FP}}$$

(4) $$\text{Accuracy}=\frac{\mathrm{TP}+\mathrm{TN}}{\mathrm{TP}+\mathrm{TN}+\mathrm{FP}+\mathrm{FN}}$$

(5) $$F1\;\text{Score}=2\times \frac{\text{Precision}\times \text{Sensitivity}}{\text{Precision}+\text{Sensitivity}}$$

Weighted average:


(6) $$P_{\text{weighted}}=\sum_{i=1}^{k}w_{i}\cdot P_{i}$$

Average precision (AP):


(7) $$\mathrm{AP}=\int_{0}^{1}p(r)\mathrm{dr}$$

Area under the receiver operating characteristic curve (AUC):


(8) $$\mathrm{AUC}=\frac{1}{2}\sum_{i=1}^{n-1}(\mathrm{FP}R_{i+1}-\mathrm{FP}R_{i})\times (\mathrm{TP}R_{i+1}+\mathrm{TP}R_{i})$$

Here, TP represents the number of samples correctly predicted as a specific BBPS category, TN denotes the number of samples correctly predicted as not belonging to that category, FP indicates the number of samples incorrectly predicted as that category, and FN refers to the number of samples incorrectly predicted as not belonging to that category. Additionally, $P_{i}$ represents the performance metric value for the i -th category, and $w_{i}$ signifies the weight assigned to the i -th category.

## Results

### Model training and validation

**Figure 6:**
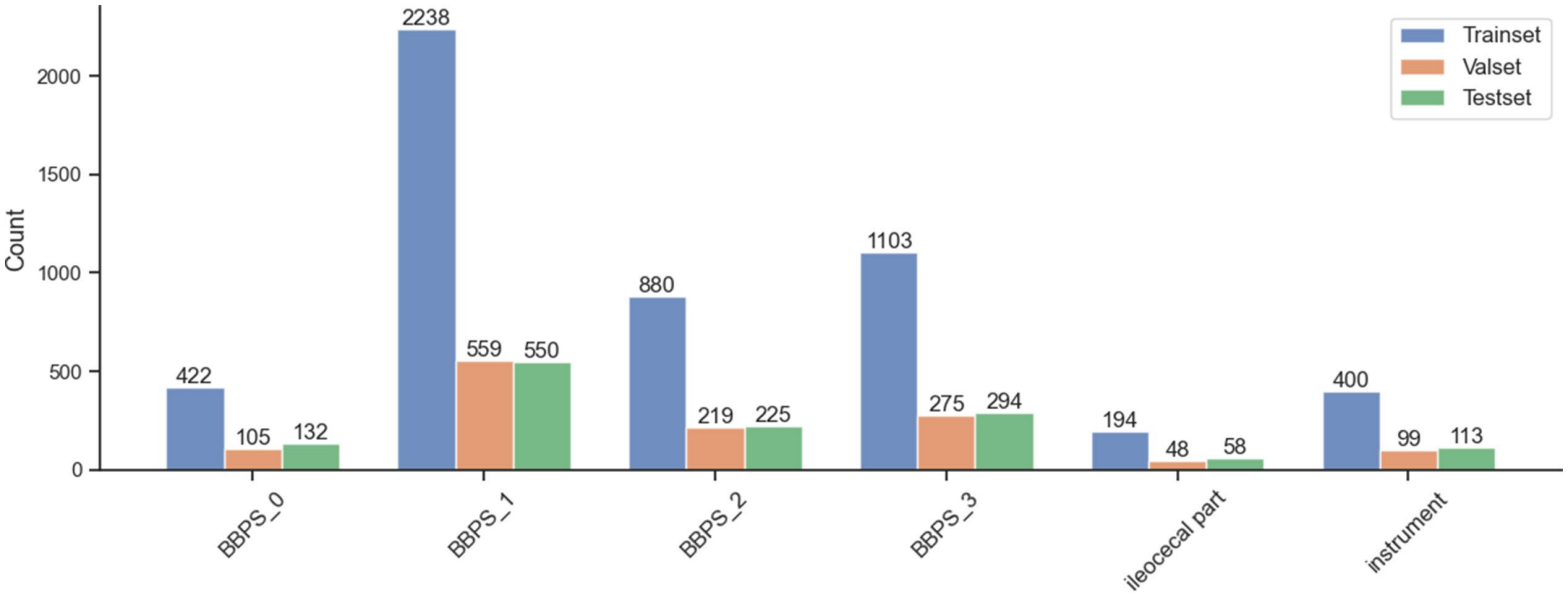
Dataset partitioning overview.

**Figure 7:**
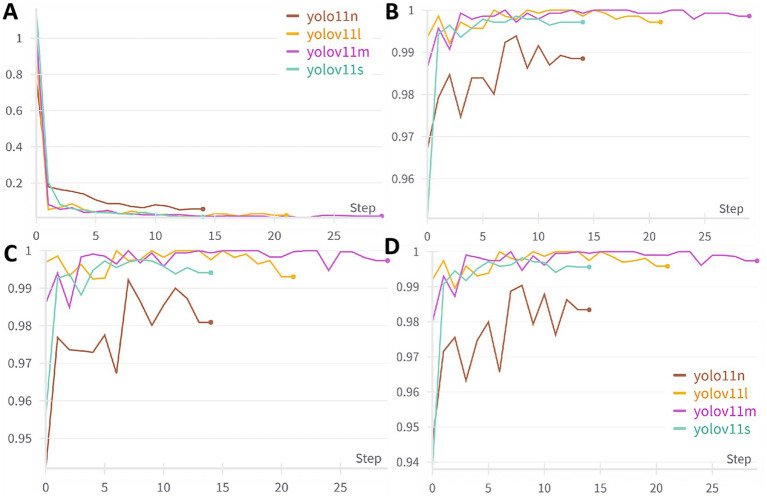
Trends in performance metrics of different models with training steps; **(A)** Loss function trends, **(B)** Accuracy trends, **(C)** Precision trends, **(D)** F1-score trends. Early stopping was applied in this study, leading to potential differences in training steps among models.

This study included a total of 7,914 images, covering six categories: BBPS 0, BBPS 1, BBPS 2, BBPS 3, ileocecal part, and instrument. Among these, 6,542 images were used for model development, while an independently collected set of 1,372 images was reserved for testing. The dataset partitioning is illustrated in Figure 6. Four YOLOv11 neural network models of varying scales—YOLOv11n, YOLOv11s, YOLOv11m, and YOLOv11l—were trained using the same dataset. The complete training process of these models was tracked using wandb. As the number of training steps increased, the model’s loss gradually decreased and stabilized, indicating convergence toward optimization (Figure 7A). Figures 7B–D depict the trends of accuracy, precision, and F1 score, respectively, across different models as training progressed. Initially, these performance metrics rose slowly with significant fluctuations but eventually stabilized at higher levels. Compared to the other three models, YOLOv11m achieved the best accuracy (99.86%), precision (99.74%), and F1 score (99.75%) on the validation set, along with the second-highest sensitivity (99.74%). Consequently, YOLOv11m was selected as the optimal model, with detailed results presented in Table 1.

**Table 1:** Performance comparison of different models on the validation set (%).

Model	Accuracy	Precision	Sensitivity	F1 score
yolov11s	99.71	99.41	99.72	99.56
yolov11l	99.71	99.31	99.88^*^	99.58
yolov11m	99.86^*^	99.74^*^	99.74	99.75^*^
yolov11n	98.81	98.78	99.08	98.56

*Indicates the best performance.

### Optimal model testing

**Figure 8:**
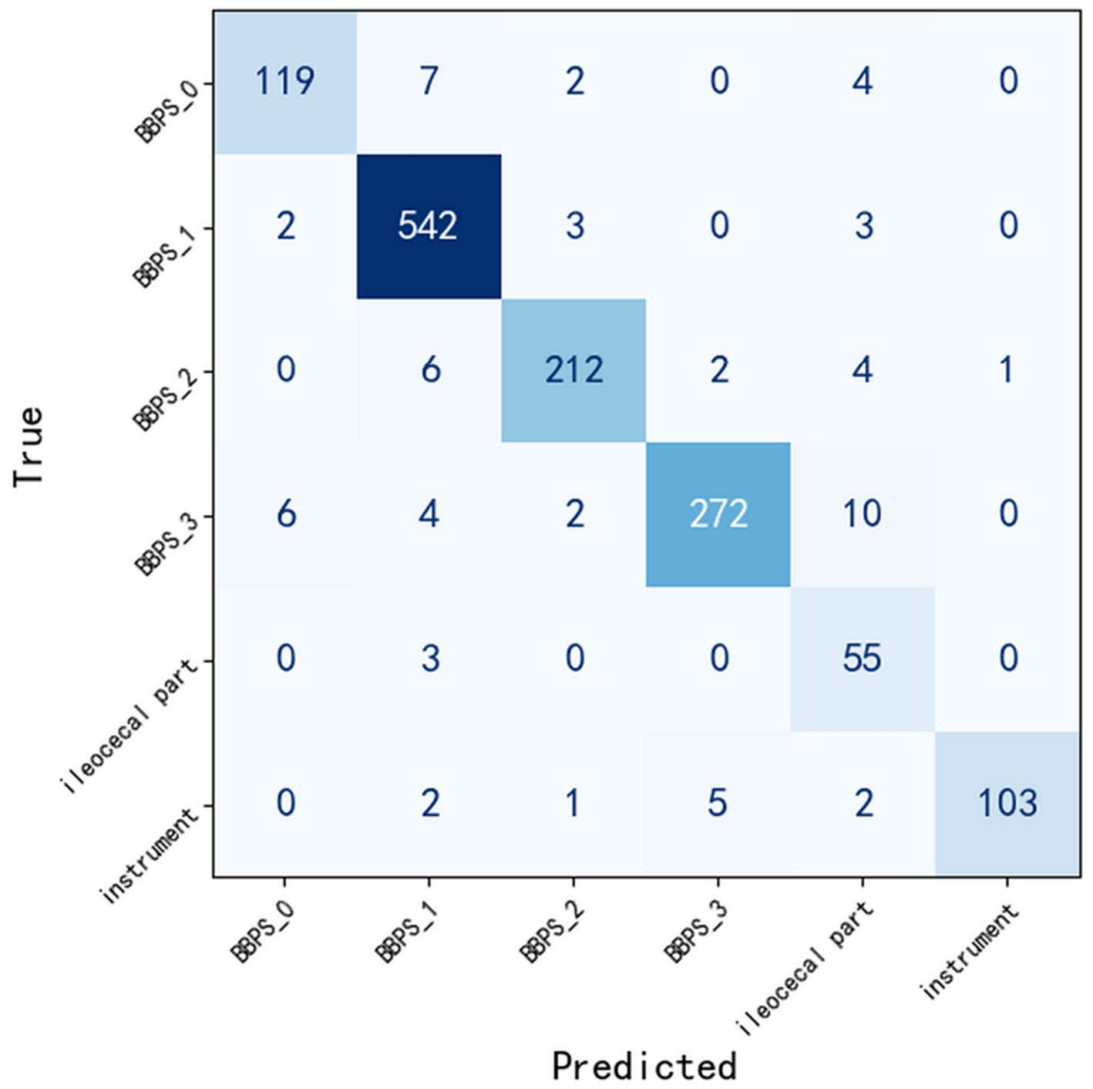
Confusion matrix of YOLOv11m model predictions on the test set.

Table 2 presents the performance of the optimal YOLOv11m model on the 1,372 test set images. The table details the model’s precision, sensitivity, specificity, F1 score, accuracy, AP, and AUC values for the six categories. Additionally, the weighted average is provided as a summary statistic, and the confusion matrix is illustrated in Figure 8.

**Table 2:** Classification performance evaluation of the YOLOv11m model on the test set.

Class	Precision %	Sensitivity %	Specificity %	F1 score %	Accuracy %	AP %	AUC
BBPS 0	93.70	90.15	99.35	91.89	98.47	91.32	0.986
BBPS 1	96.10	98.55	97.32	97.31	97.81	99.22	0.995
BBPS 2	96.36	94.22	99.3	95.28	98.47	98.04	0.996
BBPS 3	97.49	92.52	99.35	94.94	97.89	96.14	0.983
ileocecal part	70.51	94.83	98.25	80.88	98.11	94.28	0.996
instrument	99.04	91.15	99.92	94.93	99.2	96.29	0.978
Overall (weighted average)	95.37	94.97	98.53	95.06	94.97	97.16	0.990

The weighted average metric takes into account the sample size of each category, assigning higher weights to categories with larger sample sizes.

**Figure 9:**
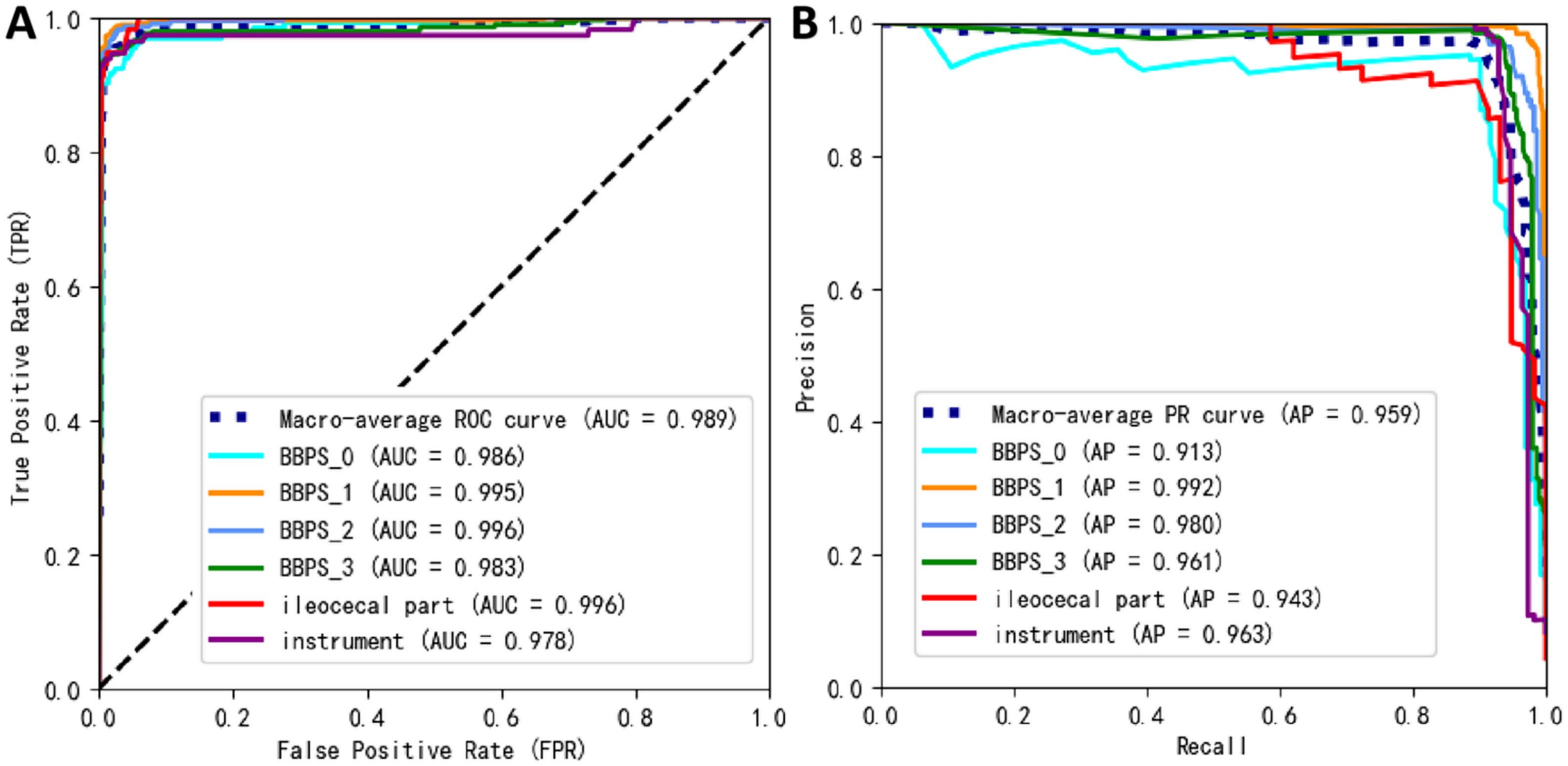
Prediction performance of YOLOv11m on the external test set; **(A)** receiver operating characteristic (ROC) curve, **(B)** precision-recall (PR) curve.

Figures 9A,B respectively illustrate two key evaluation curves for the YOLOv11m model on the test set: the Receiver Operating Characteristic (ROC) curve and the Precision-Recall (PR) curve. In Figure 9B, the ROC curves for all categories are positioned close to the top-left corner of the chart, indicating excellent model performance across these categories. In Figure 8, the PR curves demonstrate that the closer a category’s curve is to the top-right corner, the better the model’s performance for that category.

### Analysis of model interpretability

**Figure 10:**
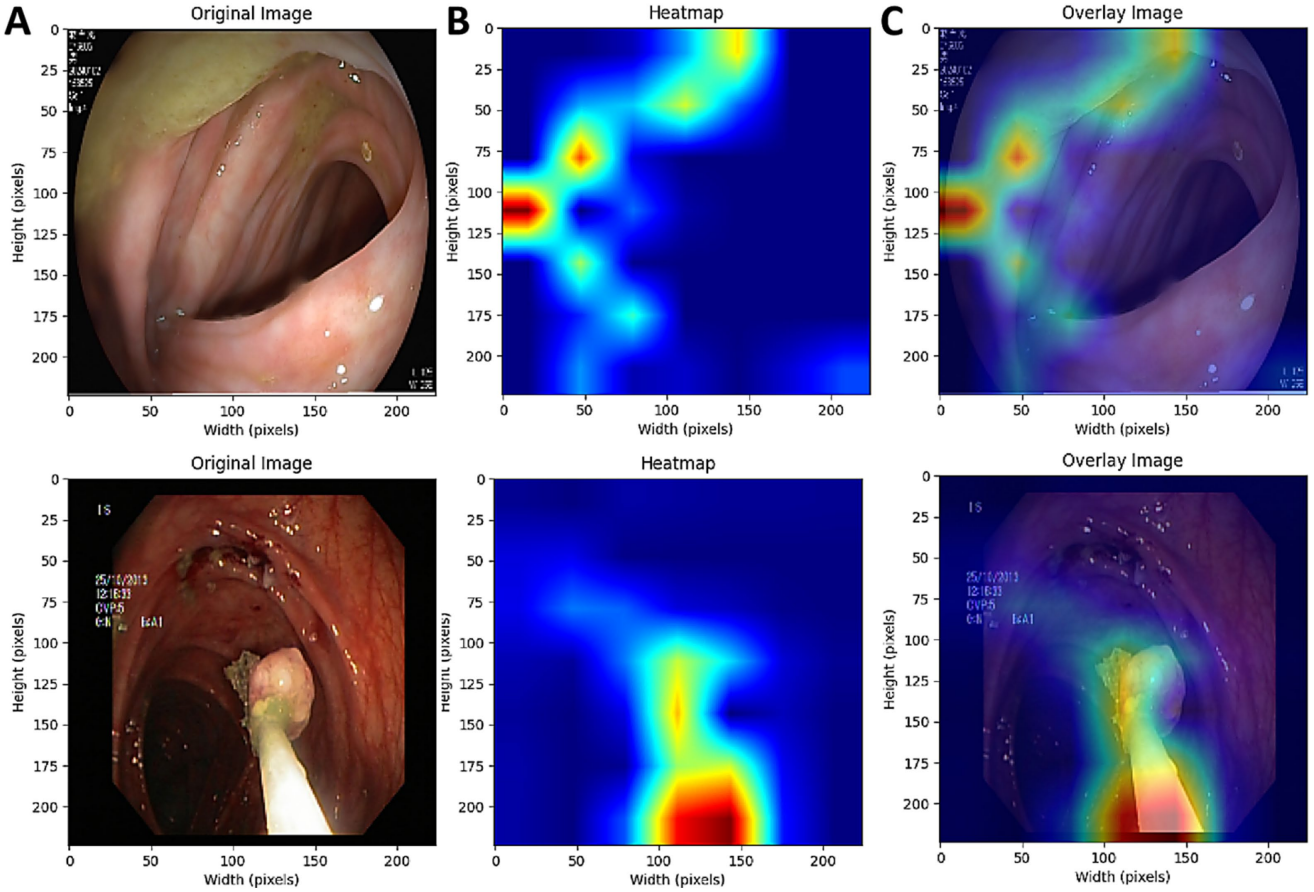
Grad-CAM visualization of the AI model’s decision-making process; column **(A)** original endoscopic images, column **(B)** pixel activation heatmaps using Grad-CAM, column **(C)** combination of original images and activation heatmaps.

Figure 10 demonstrates the visualization of the AI model’s decision-making process using Grad-CAM technology. Column A displays the original images; Column B shows the pixel activation heatmaps generated by the YOLOv11m model, highlighting the critical regions influencing the model’s decisions; Column C overlays the activation heatmaps onto the original images, with warm tones (such as red and yellow) indicating the key lesion areas identified by the model.

### Human-machine comparison

**Figure 11:**
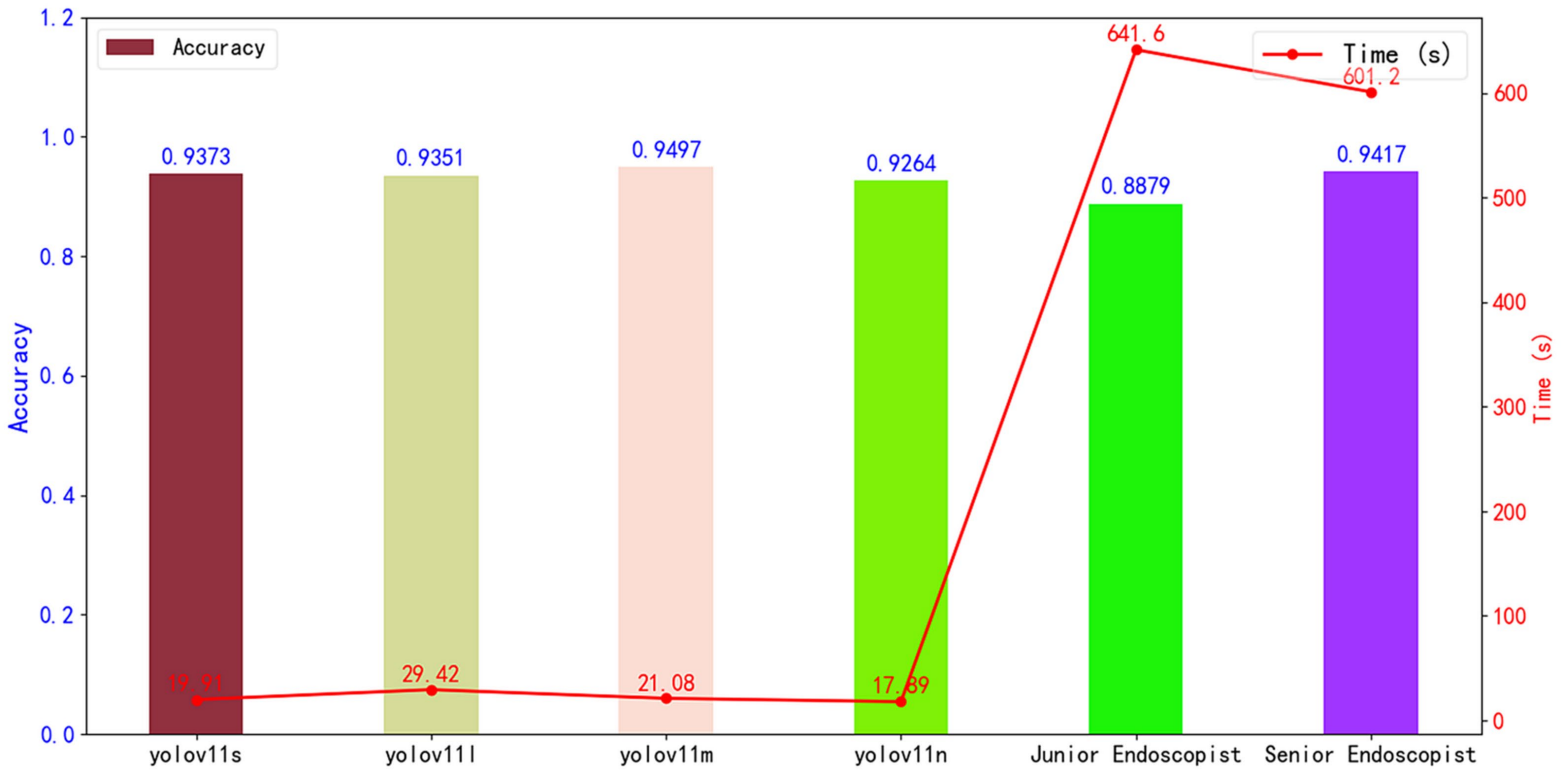
Human-AI comparison experiment (image level); the bar chart compares accuracy, while the line chart shows diagnostic time.

In the image-level human-AI comparison experiment, this study evaluated the diagnostic performance of four AI models (YOLOv11n, YOLOv11s, YOLOv11m, YOLOv11l) against endoscopists with varying levels of experience on a test set comprising 1,372 colonoscopy images (Dataset 3). The analysis focused on diagnostic accuracy and speed (measured in seconds), with results shown in Figure 11. Among all models, YOLOv11m achieved the highest diagnostic accuracy of 99.86%, significantly outperforming junior endoscopists (88.79%) and closely matching the performance of senior endoscopists (98.93%). Further χ^2^ tests revealed that the diagnostic accuracy of YOLOv11m was significantly different from that of junior endoscopists (χ^2^ = 33.49, *p* < 0.05) but not significantly different from senior endoscopists (χ^2^ = 0.57, *p* > 0.05). These results indicate that YOLOv11m surpasses less experienced junior endoscopists in diagnostic accuracy and performs comparably to highly experienced senior endoscopists. In terms of diagnostic speed, the YOLOv11n model required the least time, completing the analysis of all test images in just 17.89 s, which is approximately 35.87 times faster than junior endoscopists (641.6 s) and 33.60 times faster than senior endoscopists (601.2 s).

**Figure 12:**
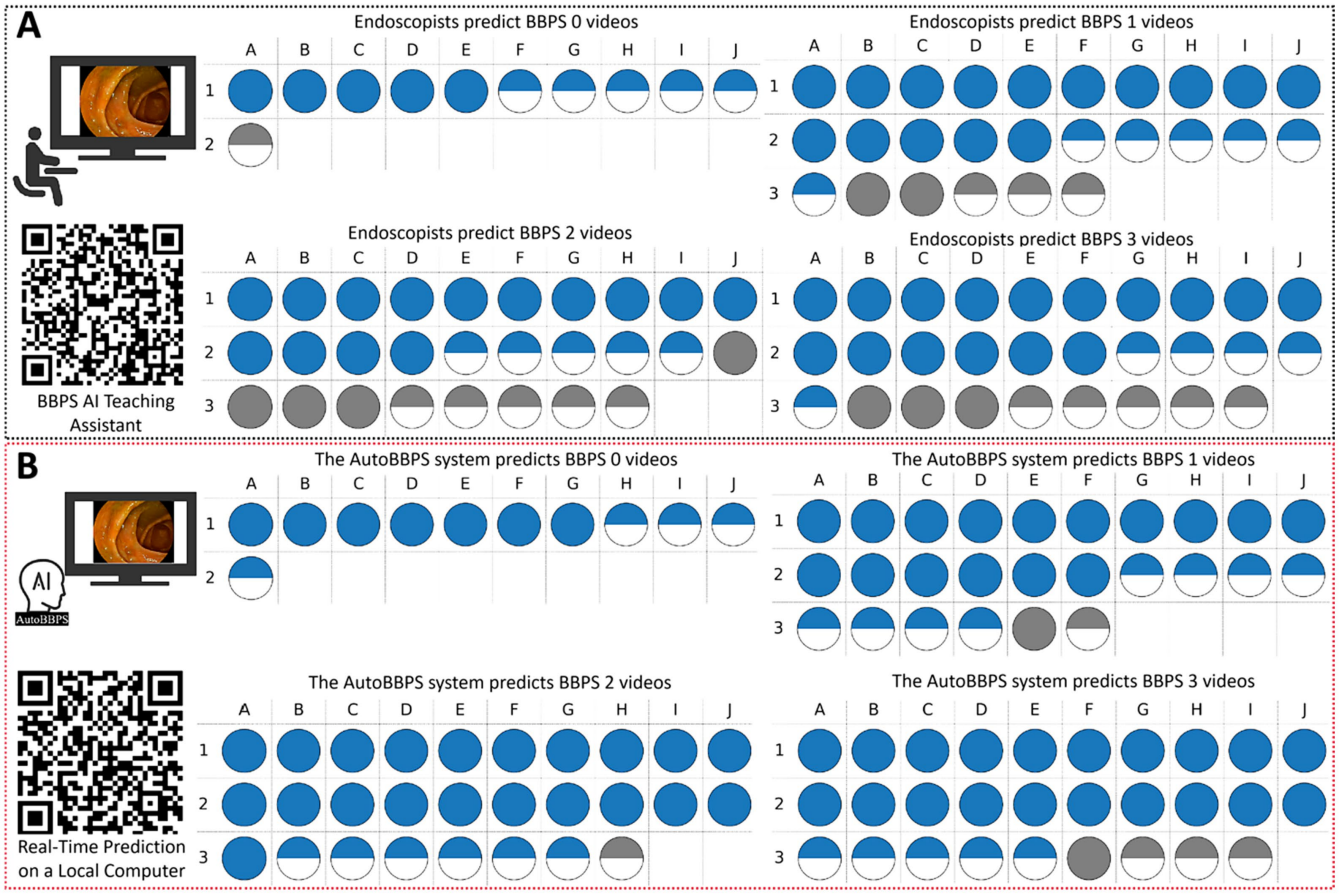
**(A)** Endoscopist predictions. **(B)** AutoBBPS predictions.

In the video-level human-AI comparison experiment, this study compared the diagnostic accuracy of the developed AutoBBPS system with that of four endoscopists at different confidence levels using a test set containing 94 colonoscopy videos (Dataset 4). The results are shown in Figure 12. Endoscopists independently evaluated all videos and recorded their judgments and confidence levels, with the averages calculated and compared to the AutoBBPS system. To enhance prediction reliability, an 80% confidence threshold was set. Dataset 4 included 94 colonoscopy videos, with 11, 26, 28, and 29 videos scoring BBPS 0, 1, 2, and 3, respectively. The results demonstrated that the AutoBBPS system outperformed the endoscopists in the number of correct predictions across all categories, with a statistically significant difference observed only in the BBPS 2 category (χ^2^ = 0.033, *p* < 0.05). Furthermore, the AutoBBPS system exhibited higher prediction reliability at high confidence levels, highlighting the system’s advantages under such conditions. Users can scan the QR code in Figure 12A to experience the BBPS intelligent teaching assistant developed in this study. Repeated use of this assistant can help gradually reduce subjective judgment differences among endoscopists. Figure 12B showcases a demonstration video of the AutoBBPS system performing real-time scoring on colonoscopy videos, which users can also watch by scanning the QR code.

## Discussion

This study utilized four different versions of YOLOv11 pre-trained models, fine-tuned using transfer learning on a dataset containing various bowel preparation cleanliness scores (BBPS), and completed model training, validation, and testing. Through performance comparison, the optimal model was selected and integrated with BBPS to successfully develop an AI-assisted system for automated BBPS scoring, named “AutoBBPS.” Upon detecting the arrival of the colonoscope at the ileocecal region, the system automatically initiates real-time cumulative BBPS scoring and excludes frames with instrument operations to enhance scoring accuracy. The clinical application value of AutoBBPS was preliminarily validated through human-AI comparison experiments conducted at both image and video levels.

BBPS is currently the most widely used tool for assessing bowel preparation cleanliness. However, studies by Zorzi et al. (15) have reported several limitations within the BBPS score range of 2 to 6: inter-rater agreement is moderate, intra-rater agreement ranges from moderate to fair, and no significant correlation has been found between BBPS and recommendations for repeat colonoscopies. Additionally, large-scale quality analyses of two colonoscopy studies found no correlation between BBPS and adenoma detection rate (ADR) (16, 17). The reasons for these limitations lie in the fact that BBPS relies on endoscopists’ subjective memory to score each of the three colon segments after completing the procedure, making it a post-procedural subjective assessment. In contrast, the AutoBBPS system provides real-time cumulative scoring during the colonoscopy by analyzing each frame of the video and objectively scoring the three colon segments. As a result, the AutoBBPS system offers greater objectivity, real-time capability, and consistency, thereby enhancing the accuracy and reliability of the scoring. Furthermore, with the advancement and widespread adoption of endoscopic minimally invasive techniques, such as endoscopic mucosal resection (EMR) and cold snare polypectomy (CSP), some eligible polyps can be removed during the initial colonoscopy. While this improves procedural efficiency, it also prolongs the operation time. The AutoBBPS system automatically excludes frames with instrument operations, saving computational resources and further improving scoring accuracy by eliminating these interfering frames.

Colonoscopy is the gold standard for colorectal cancer screening, and high-quality colonoscopy relies on adequate bowel preparation. Therefore, the evaluation of bowel preparation remains a focal point in clinical research, encompassing both pre-procedure and intra-procedure assessments. For pre-procedure evaluation, Lu et al. (16) developed an AI system based on convolutional neural networks to help patients assess bowel preparation quality at home by analyzing stool images in the toilet. Wang et al. (17) created a tool based on U-Net convolutional neural networks capable of automatically segmenting fecal regions in images. However, this tool is limited to single-frame image cleanliness assessment and cannot process colonoscopy videos in real-time or provide a comprehensive evaluation of bowel cleanliness. The AutoBBPS system developed in this study enables real-time and cumulative BBPS assessment for each frame of the video, making it more aligned with clinical needs compared to previous studies. Additionally, the system achieved prediction accuracies of 98.47, 97.81, 98.47, and 97.89% for colonoscopy images with BBPS scores of 0, 1, 2, and 3, respectively, in the test set. Furthermore, Grad-CAM technology was used to reveal the key regions identified by the model.

Studies have indicated (15) that in clinical practice, inter-rater agreement for BBPS scores ranges only from moderate to fair. This phenomenon may be attributed to the lack of systematic training and education for endoscopists, who often rely on self-learning and directly apply BBPS scoring during actual colonoscopy procedures. In this study, the optimal model was developed into a Streamlit-based mobile app. When scorers are uncertain about the images in a frame, they can use this app on their smartphones to obtain reference scoring results. This app helps advance standardized training and education, enabling junior medical staff to master the BBPS scoring method more quickly. By repeatedly using the AI teaching assistant, subjective judgment differences among medical staff can gradually be reduced, thereby supporting the optimization of bowel preparation education and management.

## Conclusions and future work

This study integrated images and videos from four datasets to develop an AI-assisted system for automated BBPS scoring and a smartphone-based intelligent teaching app. The research encompassed the entire workflow, including data collection, model training, validation, testing, interpretability analysis, terminal deployment, and human-AI comparison experiments, systematically exploring the potential of artificial intelligence in automated BBPS scoring and education.

This study used data from three medical centers employing endoscopy systems from SonoScape, Olympus, and Pentax. Validation on independent external test sets (1,372 images and 94 videos) demonstrated good cross-center and cross-vendor generalizability. The applied data augmentation strategies, such as letterbox resizing, random flipping, and color enhancement, etc., helped reduce the effects of illumination and color variations. However, the cohort was limited to a single metropolitan region without vendor- or subgroup-level stratification. Future multicenter studies across regions and vendors, incorporating device-specific analyses and appropriate model calibration or domain adaptation, are planned to further assess the system’s short- and long-term impact on BBPS scoring accuracy among endoscopists.

## Data Availability

The raw data supporting the conclusions of this article will be made available by the authors, without undue reservation.
